# Causes of death across categories of estimated glomerular filtration rate: The Stockholm CREAtinine Measurements (SCREAM) project

**DOI:** 10.1371/journal.pone.0209440

**Published:** 2019-01-16

**Authors:** Björn Runesson, Abdul R. Qureshi, Hong Xu, Alessandro Gasparini, Bengt Lindholm, Peter Barany, Carl G. Elinder, Juan J. Carrero

**Affiliations:** 1 Division of Renal Medicine and Baxter Novum, Department of Clinical Science, Technology and Intervention, Karolinska Institutet, Stockholm, Sweden; 2 Department of Medical Epidemiology and Biostatistics, Karolinska Institutet, Stockholm, Sweden; 3 Department of Health Sciences, University of Leicester, Leicester, United Kingdom; Istituto Di Ricerche Farmacologiche Mario Negri, ITALY

## Abstract

**Introduction:**

Reduced kidney function increases the risk of death, but there is limited information on causes of death across stages of chronic kidney disease (CKD). We aimed to identify leading causes of death in community-dwelling individuals with differing kidney function.

**Methods:**

Observational analysis from SCREAM, a healthcare utilization cohort of Stockholm, Sweden. We included all individuals who died during 2006–2012 and had one serum creatinine measured in the year prior to death. Using the CKD-EPI formula, we calculated eGFR and stratified individuals according to CKD stages. Causes of death were classified as cardiovascular (CVD), cancer, infection and other, using ICD-10 codes. We compared age- and sex-adjusted differences in the proportions of deaths from each cause.

**Results:**

Out of 89,117 registered deaths, 70,547 (79%) had a recent eGFR estimation and were included in this study. Individuals had a median age of 82 (IRE 62–93) years and 52% were women. The proportions of deaths from CVD increased with lower eGFR, along with the proportion of deaths from infections. Deaths due to diabetes and genito-urinary diseases increased. Deaths due to cancer decreased, but other death causes did not vary. Within CVD causes of death, the proportion of arrhythmias and heart failure increased, but ischemic heart disease and cerebrovascular disease remained stable.

**Conclusion:**

In a region-representative Swedish healthcare extraction, we observe differences regarding specific causes of death across different CKD stages. Increasing patient and provider awareness of this differential pattern of risk may have benefits for patient management, prevention strategies, and health service planning.

## Introduction

Chronic kidney disease (CKD) is a common but under-recognized health problem, affecting 5–10% of the European population, especially the elderly and the chronically ill [1, 2]. While it is well-known that low kidney function increases mortality [3–6], the pattern of causes of death associated with CKD stages has not been sufficiently described. Characterizing causes of death in the community in relation to kidney function is important, since the prevention and management of underlying diseases can be substantially different across increasing disease severity.

Available reports on this issue are scarce and derived from North American [4, 7], or Korean populations [6]. Because of differences in lifestyle, risk factors, clinical practice patterns, and healthcare access, comparisons with other countries and regions may be useful as a measure of the overall disease burden. Potentially, it may also serve to generate insights into mechanisms of disease and help to identify population segments where more intensive preventive strategies are needed. To this end, we used a large region-representative healthcare-extraction to examine the proportions of deaths attributable to various causes in people at varying levels of kidney function in Stockholm, Sweden.

## Material and methods

### Data source and study design

This study is based on the Stockholm CREAtinine Measurements (SCREAM) healthcare-utilization cohort [1], including all residents in the region of Stockholm, Sweden, that undertook at least one measurement of serum creatinine in ambulatory or hospital care during 2006–2011. Laboratory data were linked with regional and national administrative databases for complete information on healthcare utilization, dispensed drugs, validated renal replacement therapy endpoints and follow up for death. This repository captured 89% of deaths occurring during 2006–2011 in the Stockholm county, with non-captured deaths attributed to younger population segments (<65 years old) who less often accessed healthcare or had creatinine tested [1].

For this analysis, we identified adults aged ≥18 years old who were residing in Stockholm, died between January 1^st,^ 2006 and March 31^st,^ 2012, and had an outpatient serum creatinine measured in the year before death. Stockholm is the most populated region in Sweden, with a population of 1.8 million individuals in 2011. In Sweden, there is universal access to the publicly financed healthcare, which is provided by both public and private institutions. The institutional review board at Karolinska Institutet, Stockholm, Sweden and the Swedish National Board of Welfare approved the study.

### Exposure and covariates

All creatinines were measured with either enzymatic or corrected Jaffe method (alkaline picrate reaction), both methods being traceable to isotope dilution mass spectroscopy standards. Inter- as well as intra-laboratory variation is considered minimal in the region, with the three laboratories being frequently audited for quality and harmonization by the national organization EQUALIS (www.equalis.se). For this study, we excluded creatinine laboratory tests measured at hospital and at the emergency department, as well as implausible values (<40 and >1500 mmol/L). We selected all remaining serum creatinine determinations >1 day before and within the year prior to death. If more than one eligible creatinine was available for a patient, we used the one closest to the time of death.

We used these serum creatinine measurements to determine the exposure, which is estimated glomerular filtration rate (eGFR) strata, using the 2009 CKD-EPI creatinine-based equation. Records of ethnicity are not available in Sweden, and in applying this equation, all individuals were considered Caucasians. Using the value closest in time, and up to one year before death, outpatient eGFR was categorized as >90, 90–60, 45–59.9, 30–44.9, 15–29.9 ml/min per 1.73 m^2^, and end-stage renal disease (ESRD). The category ESRD included individuals with eGFR <15 ml/min per 1.73 m^2^ as well as those undergoing maintenance dialysis (hemodialysis or peritoneal dialysis). Individuals with a renal transplant where assigned to the eGFR category of their eligible creatinine. Renal replacement therapy status was ascertained by linkage with the Swedish Renal Register (http://www.snronline.se). Because albuminuria was only available in 13% of the cohort [1], it could infer selection bias; we were therefore unable to study KDIGO CKD severity stages [8], and in this study we refer solely to eGFR strata.

All other covariates were calculated at the time of the index eGFR. Demographic variables included age at time of the index eGFR, and sex. We used validated algorithms to define the Charlson comorbidities at baseline using ICD-10 diagnoses issued in the preceding 5 years in connection to hospitalization and ambulatory care [9].The Charlson Comorbidity Index score included the following 16 comorbidity domains: myocardial infarction, congestive heart failure, peripheral vascular disease, cerebrovascular disease, dementia, chronic pulmonary disease, rheumatic disease, mild liver disease, diabetes without chronic complication, diabetes with chronic complication, hemiplegia or paraplegia, renal disease, any malignancy, moderate or severe liver disease, metastatic solid tumor, aids/HIV. For Cardiovascular disease was defined as the composite of myocardial infarction, congestive heart failure, peripheral vascular disease and cerebrovascular disease.

### Study outcome

The outcome was causes of death, as registered by the Swedish Population Register, which analyses death records and classifies underlying medical conditions according to the International Classification of Disease-10^th^ revision (ICD-10). The population is updated monthly by the Swedish National Board of Welfare and is considered to have virtually no loss to follow up. For this study, we considered the primary assigned cause of death, which is coded to ICD-10 diagnoses from the written death certificates by the treating physician. Causes of death were divided into four main categories: cardiovascular, cancer, infection and other. Other causes included neurologic diseases, chronic lung diseases, digestive diseases, accidents (suicides included), diabetic complications, and miscellaneous (any cause not fitting the above). There were no missing causes of death. Cardiovascular causes of death were subdivided into ischemic heart disease, congestive heart failure, cerebrovascular disease, arrhythmia and valvular disease.

### Statistical analyses

We report baseline descriptive statistics as counts and percentages or medians and interquartile ranges as appropriate. Chi-squared and Kruskal–Wallis tests were used to test for differences across groups. Using multinomial logistic regression models, we estimated likelihood of death from specific causes according to eGFR strata. We reported crude and age- and sex-adjusted proportions of death by the different causes. We also present the subcategories associated with cardiovascular and other causes of death. In a sensitivity analyses, we excluded patients with eGFR values from the last 3 months before death in an attempt to eliminate influence of critical illness unrelated to chronic kidney dysfunction. Finally, we analyzed causes of death among men and women separately. Analyses were performed using Stata 14.2 (Stata Corp LLC, 4095 Lakeway drive, College Station, Tx 77846, USA) and SAS version 9.4 (SAS Campus Drive, Cary, NC, USA).

## Results

### Study flow-chart and participant characteristics

**Fig 1:**
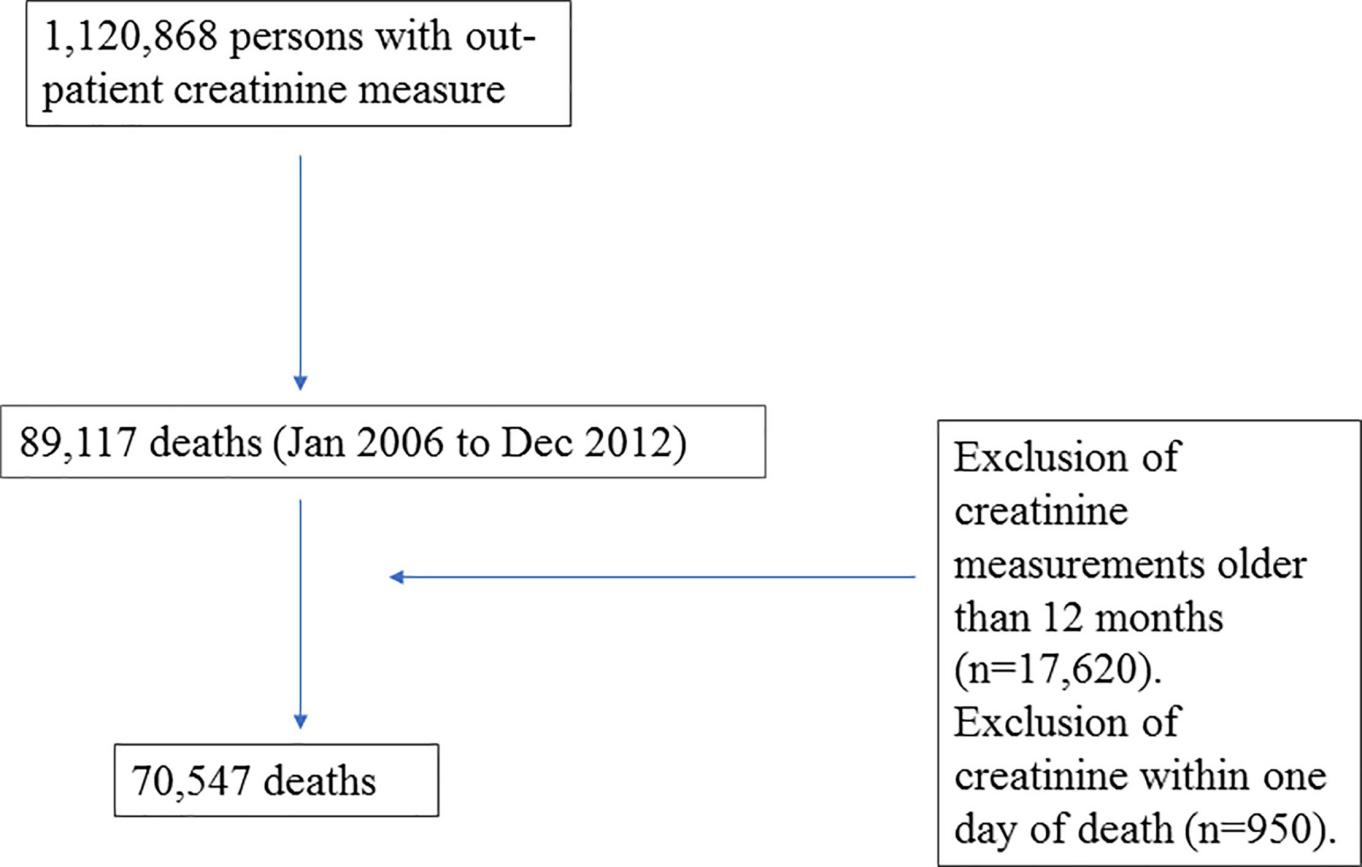
Study patient selection flow chart.

During the study period, a total of 1.120.868 adult healthcare users underwent creatinine testing in Stockholm. Of these, 89.117 individuals died. We excluded 17.620 of them because their closest ambulatory creatinine to calculate eGFR was taken more than 12 months prior to death. Further 950 individuals were excluded because the index creatinine was measured on the day of or the day prior to death (and presumably impacted by acute disease). At the end of the selection process, a total of 70.547 cases of death were included in the study (Fig 1).

Table 1 shows baseline characteristics of the study participants overall and according to the four main categories of death. The median age was 82 years (10^th^ and 90^th^ percentiles 62–93 years) and 52% were women. A diagnosis of diabetes was found among 18% of participants, and 44% had a history of CVD. CVD was the most common cause of death (36% of deaths, n = 25,216), followed by cancer (31%, n = 21,941). Participants dying from cancer were younger and less often diabetic as compared to those dying from CVD or infection. Between 50 and 60% of cases, depending on the specific cause of death, had an eGFR>60 ml/min.

**Table 1:** Demographic and clinical characteristics of study participants, overall and by cause of death.

Participant Characteristics	Overall	Death attributed to			
		CVD	Cancer	Infection	Other
N (%)	70 547 (100)	25,216 (35.7)	21,941 (31.1)	3250 (4.6)	20,140 (28.55)
Age, years (median, 10^th^ to 90^th^ p)	82 (62 to 93)	86 (69 to 94)	75 (57 to 88)	83 (60 to 93)	84 (62 to 94)
Women, N (%)	36,878 (52.3)	13,573 (53.8)	10,649 (48.5)	1612 (49.6)	11044 (54.8)
Diabetes, N (%)	12,855 (18)	4,946 (19.6)	3,407 (15.5)	639 (19.7)	3,863 (19.2)
CVD, N (%)	31,393 (44.5)	15,417 (61.1)	5,986 (27,28)	1646 (50.7)	8,344 (41.4)
Charlson score (median, 10^th^ to 90^th^ p)	2 (0 to 8)	2 (0 to 5)	5 (1 to 9)	2 (0 to 5)	2 (0 to 5)
**eGFR strata, ml/min /1.73 m**^**2**^					
>90, N (%)	12,538(17.8)	2,171 (8.6)	6,503 (29.6)	396 (12.2)	3,468 (17.2)
60–89, N (%)	28,937 (41.0)	10,046 (39.8)	9,213 (42)	1,241 (38.2)	8,437 (41.9)
45–59, N (%)	12,260 (17.4)	5,227 (20.7)	3,016 (13.8)	652 (20.0)	3,365 (16.7)
30–44, N (%)	9,868 (14)	4,603 (18.3)	2,001 (9.1)	582 (17.9)	2,682 (13.3)
15–29, N (%)	5,229 (7.4)	2,488 (9.9)	965 (4.4)	292 (9.0)	1,484 (7.4)
ESRD	1,715 (2.4)	681 (2.7)	243 (1.1)	87 (2.7)	704 (3.5)

CVD, cardiovascular disease; eGFR, estimated glomerular filtration rate; ESRD, end-stage renal disease.

### Main causes of death by eGFR strata

**Fig 2:**
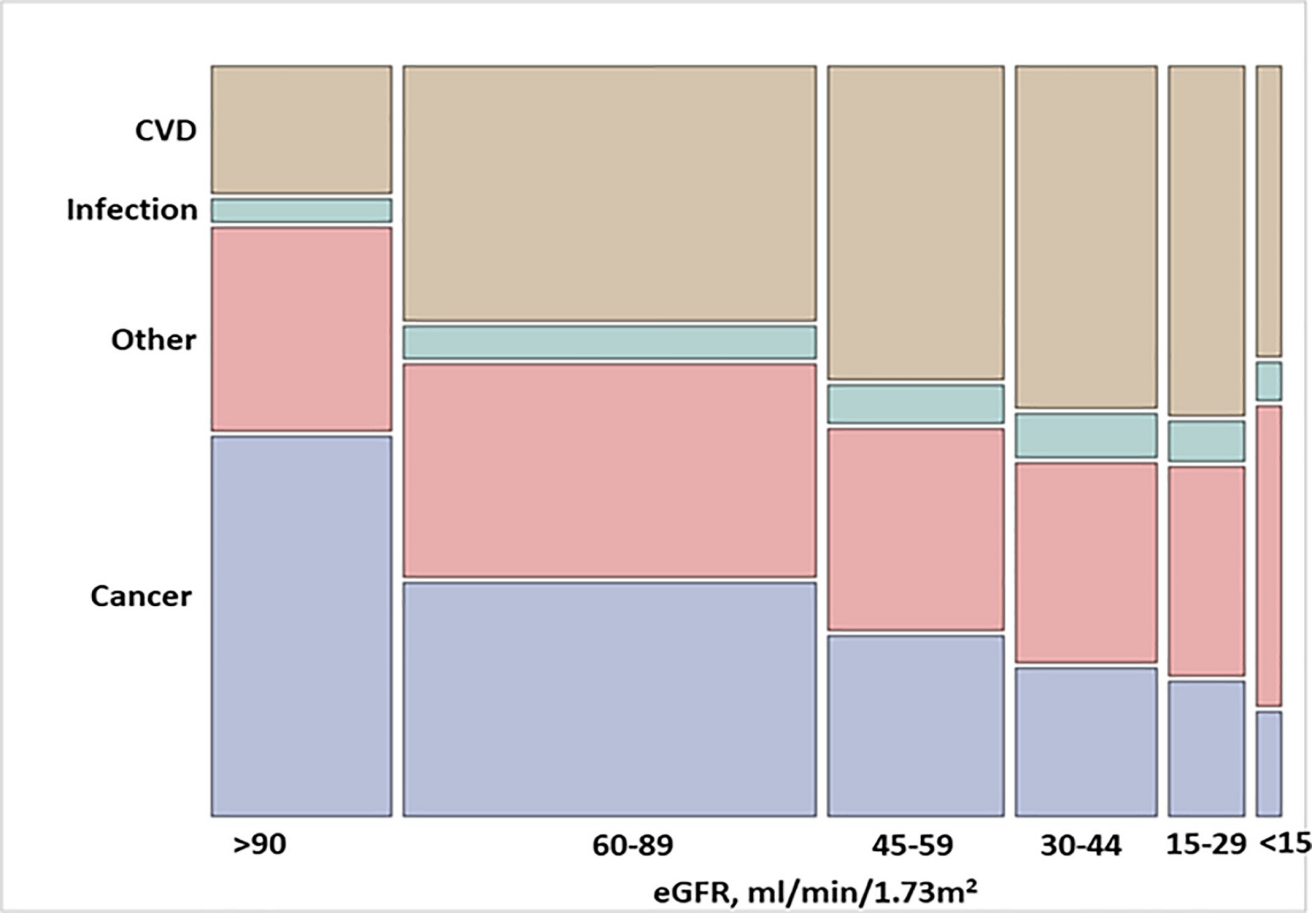
Unadjusted proportions of main causes of death by eGFR strata. The height of each colored bar represents the percentage of participants for each cause of death within each category of eGFR. The width of the bar represents the percentage of participants for each eGFR category within each cause of death. The area of each colored bar represents the percentage of participants within each eGFR category and each cause of death. CVD, cardiovascular disease.

Fig 2 shows the (unadjusted) proportions of eGFR strata according to causes of death. Table 2 shows the unadjusted and age and sex adjusted proportions of death by cause and eGFR strata. Among those with an eGFR> 90 ml/min/1.73 m^2^, cancer was the most common cause, responsible for 52% of deaths (95% CI, 51–53%). After age and sex adjustment, cancer remained the most common cause of death in this category (47% (95% CI 46–48%), followed by other causes (adjusted proportion 29% (95% CI 28–30%). Among participants with eGFR 60–89 ml/min/1.73 m^2^, deaths attributed to cancer, CVD and other causes were similar in proportion and each approximated 30% of cases.

**Table 2:** Unadjusted and age- and sex- adjusted percentages (95% CIs) of deaths by cause and eGFR strata.

	Death attributed to			
eGFR strata	CVD	Cancer	Infection	Other
**>90 ml/min/1.73 m**^**2**^				
Unadjusted	17.3 (16.7 to 18.0)	51.9 (51.0 to 52.7)	3.2 (2.9 to 3.5)	27.7 (26.9 to 28.4)
Age/sex adjusted	21.2 (20.3 to 22.0)	46.6 (45.6 to 47.6)	3.5 (3.2 to 3.9)	28.7 (27.8 to 29.6)
**60–89 ml/min/1.73 m**^**2**^				
Unadjusted	34.7 (34.3 to 35.2)	31.8 (31.3 to 32.4)	4.3 (4.1 to 4.5)	29.2 (28.6 to 29.7)
Age/sex adjusted	33.5 (33.0 to 34.1)	32.7 (32.2 to 33.3)	4.3 (4.0 to 4.5)	29.5 (29.0 to 30.0)
**45–59 ml/min/1.73 m**^**2**^				
Unadjusted	42.6 (41.8 to 43.5)	24.6 (23.8 to 25.4)	5.3 (4.9 to 5.7)	27.4 (26.7 to 28.2)
Age/sex adjusted	40.8 (39.9 to 41.7)	26.0 (25.2 to 26.8)	5.3 (4.9 to 5.7)	27.8 (27.0 to 28.7)
**30–44 ml/min/1.73 m**^**2**^				
Unadjusted	46.6 (45.7 to 47.6)	20.3 (19.5 to 21.1)	5.9 (5.4 to 6.4)	27.2 (26.3 to 28.1)
Age/sex adjusted	44.7 (43.7 to 45.7)	21.6 (20.8 to 22.5)	5.9 (5.4 to 6.4)	27.7 (26.8 to 28.6)
**15–29 ml/min/1.73 m**^**2**^				
Unadjusted	47.6 (46.2 to 48.9)	18.5 (17.4 to 19.5)	5.6 (5.0 to 6.2)	28.4 (27.2 to 29.6)
Age/sex adjusted	45.8 (44.5 to 47.2)	19.6 (18.5 to 20.7)	5.6 (5.0 to 6.2)	29.0 (27.7 to 30.0)
**ESRD**				
Unadjusted	39.7 (37.4 to 42.0)	14.2 (12.5 to 15.8)	5.1 (4.0 to 6.1)	41.0 (38.7 to 43.4)
Age/sex adjusted	38.9 (36.5 to 41.2)	14.2 (12.6 to 15.9)	5.0 (4.0 to 6.0)	41.9 (39.6 to 44.3)
**Total (cases)**	25216	21941	3250	20140

CVD, cardiovascular disease; eGFR, estimated glomerular filtration rate; ESRD, end-stage renal disease.

Among participants with eGFR<60 ml/min/1.73 m^2^, CVD appeared as the primary cause of death, with increasing proportion across lower eGFR strata (adjusted proportions of 41%, 45% and 46% of cases in the categories of eGFR 45–59, 30–44 and 15–29 ml/min/1.73 m^2^, respectively). Among ESRD cases, CVD deaths (adjusted proportion 39% (95% CI 36.5–41.2%) and other causes of death (42% (95% CI 39.6–44.3%) were of similar magnitude. Deaths due to infections were also higher in participants with eGFR<60 ml/min/1.73 m^2^, with no detectable trends across lower eGFR strata.

#### Other causes of death by eGFR strata

**Fig 3:**
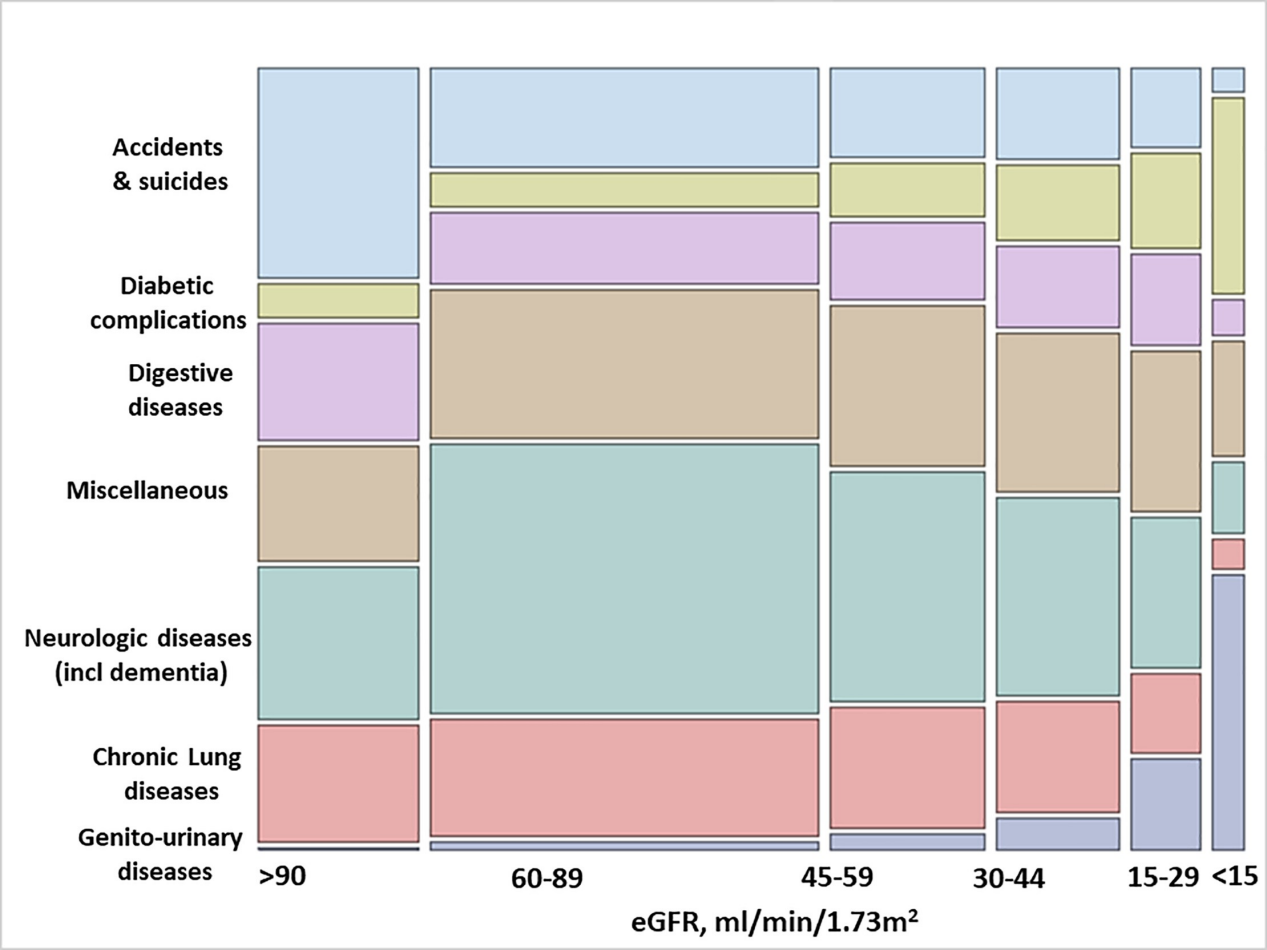
Unadjusted proportions of other causes of death by eGFR strata. The height of each colored bar represents the percentage of participants for each cause of death within each category of eGFR. The width of the bar represents the percentage of participants for each eGFR category within each cause of death. The area of each colored bar represents the percentage of participants within each eGFR category and each cause of death.

Other causes of death are presented in Table 3 and Fig 3. Neurologic diseases (including dementia), chronic lung disease and accidents/suicide were the most common causes of death among individuals with eGFR>90 ml/min/1.73 m^2^, but their proportions decreased across lower eGFR strata (for instance, from an adjusted proportion for neurologic disease of 31% (95% CI 29.2–33.2%) in eGFR>90 ml/min/1.73m2 to 9% (95% CI 7.1–11.4%) in ESRD participants). The proportion of deaths attributed to digestive diseases were similar across all eGFR strata. Conversely, minor causes of death, such as diabetic complications, genitourinary diseases and miscellaneous causes, became increasingly more common among individuals with lower eGFR.

**Table 3:** Unadjusted and age- and sex-adjusted proportions (with 95% CI) of deaths attributed to other causes by eGFR strata.

	Deaths attributed to						
eGFR. ml/min per 1.73	Neurologic diseases (incl dementia)	Chronic Lung diseases	Accidents and suicides	Digestive diseases	Diabetic complications	Genito-urinary diseases	Miscellaneous
**>90 ml/min/1.73 m**^**2**^							
Unadjusted	20.2 (18.9 to 21.6)	15.6 (14.4 to 16.8)	28.2 (26.7 to 29.7)	15.6 (14.4 to 16.8)	4.4 (3.8 to 5.1)	0.40 (0.20 to 0.61)	15.5 (14.3 to 16.7)
Age/sex adjusted	31.2 (29.2 to 33.2)	21.2 (19.5 to 22.8)	15.5 (14.1 to 16.9)	12.1 (10.9 to 13.4)	3.9 (3.2 to 4.6)	0.47 (0.22 to 0.72)	15.6 (14.2 to 17.0)
**60–89 ml/min/1.73 m**^**2**^							
Unadjusted	35.9 (34.8 to 36.9)	15.7 (15.0 to 16.5)	13.3 (12.6 to 14.0)	9.5 (8.9 to 10.2)	4.5 (4.1 to 5.0)	1.1 (0.89 to 1.4)	19.9 (19.1 to 20.1)
Age/sex adjusted	32.6 (31.5 to 33.7)	15.2 (14.4 to 16.0)	14.7 (13.9 to 15.5)	10.2 (9.5 to 10.9)	4.8 (4.4 to 5.3)	1.1 (0.85 to 1.3)	21.4 (20.5 to 22.3)
**45–59 ml/min/1.73 m**^**2**^							
Unadjusted	30.8 (29.2 to 32.3)	16.2 (14.9 to 17.4)	11.9 (10.8 to 12.9)	10.3 (9.3 to 11.3)	7.3 (6.4 to 8.2)	2.3 (1.8 to 2.8)	21.4 (20.0 to 22.8)
Age/sex adjusted	26.8 (25.3 to 28.4)	15.2 (14.0 to 16.5)	13.7 (12.4 to 15.0)	11.3 (10.2 to 12.5)	7.9 (6.9 to 8.9)	2.2 (1.7 to 2.7)	22.8 (21.3 to 24.3)
**30–44 ml/min/1.73 m**^**2**^							
Unadjusted	26.7 (25.0 to 28.4)	14.8 (13.5 to 16.2)	12.3 (11.0 to 13.5)	10.8 (9.6 to 12.0)	10.0 (8.9 to 11.2)	4.2 (3.5 to 5.0)	21.1 (19.6 to 22.7)
Age/sex adjusted	22.8 (21.2 to 24.4)	13.8 (12.5 to 15.1)	14.3 (12.9 to 15.7)	11.9 (10.6 to 13.2)	10.9 (9.6 to 12.1)	4.0 (3.3 to 4.8)	22.2 (20.1 to 23.9)
**15–29 ml/min 1.73 m**^**2**^							
Unadjusted	20.0 (18.0 to 22.0)	10.5 (9.0 to 12.1)	10.7 (9.1 to 12.3)	12.3 (10.6 to 13.9)	12.6 (10.9 to 14.3)	12.3 (10.7 to 12.0)	21.6 (19.5 to 23.7)
Age/sex adjusted	17.4 (15.5 to 19.2)	9.8 (8.3 to 11.3)	12.0 (10.3 to 13.8)	13.1 (11.3 to 14.9)	13.4 (11.6 to 15.1)	11.7 (10.0 to 13.4)	22.6 (20.4 to 22.8)
**ESRD**							
Unadjusted	9.5 (7.3 to 11.7)	4.0 (2.5 to 5.4)	3.3 (2.0 to 4.6)	4.8 (3.2 to 64)	26.1 (23.0 to 29.4)	36.9 (33.4 to 40.5)	15.3 (12.7 to 18.0)
Age/sex adjusted	9.3 (7.1 to 11.4)	4.0 (2.5 to 5.4)	3.1 (1.9 to 4.4)	4.9 (3.3 to 6.5)	26.8 (23.4 to 30.1)	35.5 (31.9 to 39.2)	16.4 (13.6 to 19.2)
**Total (cases)**	5843	2994	3012	2198	1421	740	3932

#### Subcategories of cardiovascular causes of death by eGFR strata

**Fig 4:**
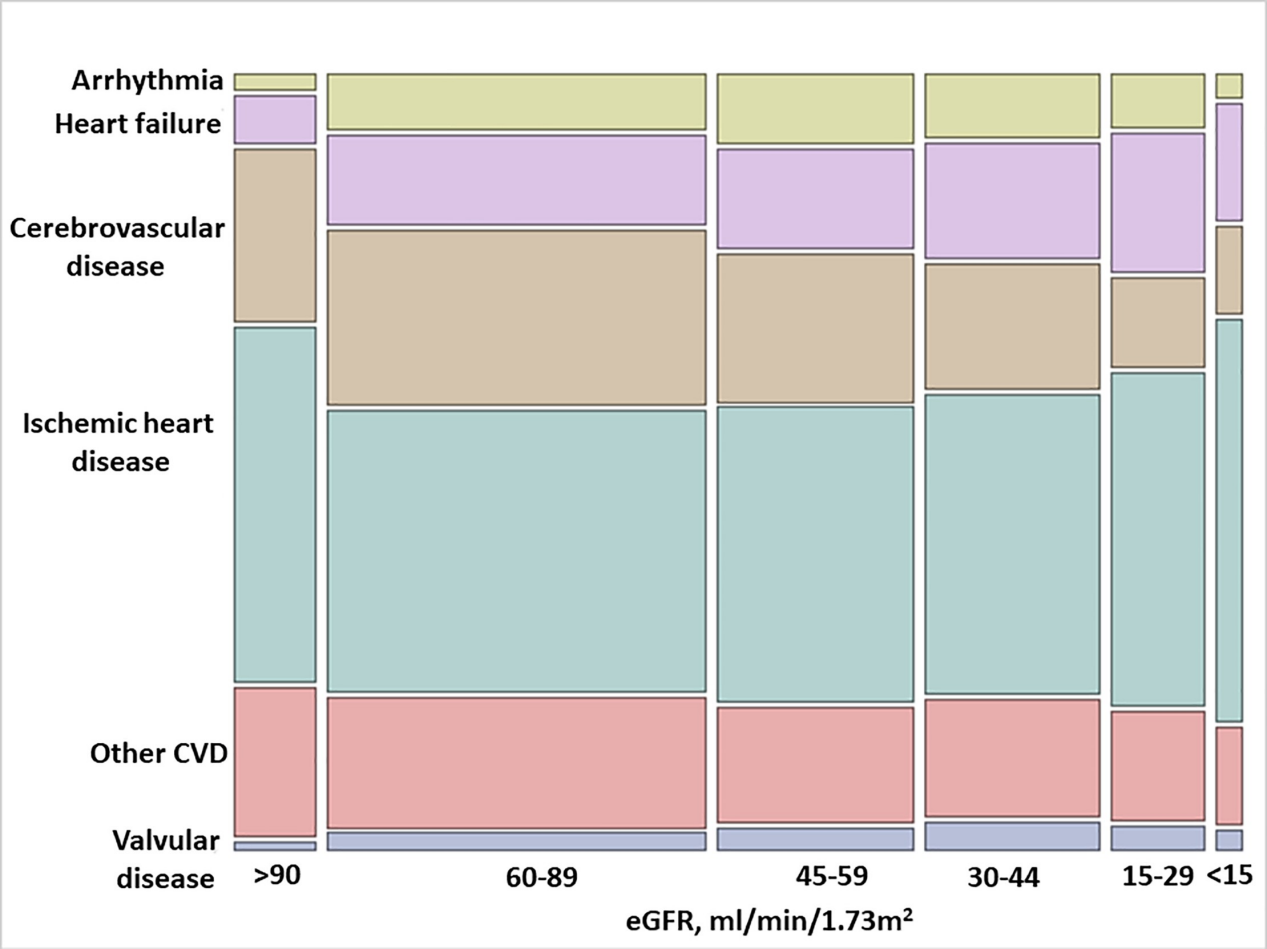
Unadjusted proportions of cardiovascular causes of death by eGFR strata. The height of each colored bar represents the percentage of participants for each cause of death within each category of eGFR. The width of the bar represents the percentage of participants for each eGFR category within each cause of death. The area of each colored bar represents the percentage of participants within each eGFR category and each cause of death. CVD, cardiovascular disease.

Fig 4 and Table 4 show a subclassification of cardiovascular causes of death by eGFR strata. Ischemic heart disease was the most common cardiovascular cause of death across all eGFR strata. Deaths attributed to cerebrovascular disease were the second most common cause of CVD-death among individuals with eGFR>90 ml/min/1.73 m^2^, but their proportion progressively decreased with lower eGFR. Conversely, deaths attributed to heart failure, valvular disease and arrhythmias tended to be more common in participants with lower eGFR strata. The proportion of other (unclassified) CVD causes of death tended to slightly decrease with lower eGFR.

**Table 4:** Unadjusted and age and sex adjusted proportions (with 95% CI) of deaths attributed to cardiovascular causes by eGFR strata.

	Deaths attributed to					
eGFR strata	Ischaemic heart Disease	Cerebrovascular disease	Heart Failure	Arrythmia	Valvular disease	Other CVD causes
**>90 ml/min/1.73 m**^**2**^						
Unadjusted	47.5 (45.4 to 49.6)	23.0 (21.2 to 24.7)	6.3 (5.3 to 7.3)	2.1 (1.5 to 2.7)	1.4 (0.9 to 1.9)	19.7 (18.0 to 21.4)
Age/sex adjusted	41.6 (34.2 to 50.0)	27.3 (22.2 to 32.4)	8.9 (6.8 to 11.0)	2.8 (-13.7 to 19.4)	1.3 (0.8 to 1.9)	18.0 (14.5 to 21.6)
**60–89 ml/min/1.73 m**^**2**^						
Unadjusted	37.6 (36.6 to 38.5)	23.3 (22.5 to 24.1)	11.8 (11.2 to 12.4)	7.5 (7.0 to 8.0)	2.5 (2.2 to 2.8)	17.3 (16.6 to 18.1)
Age/sex adjusted	38.1 (22.2 to 54.0)	23.4 (13.6 to 33.2)	11.3 (6.5 to 16.0)	7.0 (-32.0 to 46.0)	2.5 (1.4 to 3.6)	17.8 (10.3 to 25.1)
**45–59 ml/min/1.73 m**^**2**^						
Unadjusted	39.2 (37.9 to 40.5)	19.5 (18.4 to 20.6)	13.2 (12.3 to 14.1)	9.4 (8.6 to 10.2)	3.4 (2.9 to 3.9)	15.3 (14.3 to 16.3)
Age/sex adjusted	40.7 (19.9 to 61.5)	19.3 (9.4 to 29.2)	12.2 (5.9 to 19.0)	8.5 (-38.4 to 55.5)	3.5 (1.7 to 5.4)	15.8 (7.7 to 23.8)
**30–44 ml/min/1.73 m**^**2**^						
Unadjusted	39.8 (38.4 to 41.3)	16.7 (15.6 to 16.7)	15.1 (14.1 to 16.1)	8.5 (7.7 to 9.3)	4.4 (3.8 to 5.0)	15.5 (14.4 to 16.5)
Age/sex adjusted	41.3 (22.2 to 60.4)	16.5 (8.8 to 24.1)	14.0 (7.5 to 20.5)	7.7 (-35 to 50.5)	4.6 (2.4 to 6.7)	16.0 (8.5 to 23.4)
**15–29 ml/min 1.73 m**^**2**^						
Unadjusted	44.2 (42.3 to 46.2)	11.9 (10.6 to 13.2)	18.3 (16.8 to 19.8)	7.2 (6.2 to 8.2)	4.0 (3.2 to 4.8)	14.4 (13.0 to 15.8)
Age/sex adjusted	45.7 (27.6 to 63.8)	11.8 (7.0 to 16.6)	17.1 (10.2 to 23.9)	6.6 (-30.5 to 43.7)	4.1 (2.3 to 5.9)	14.8 (8.8 to 20.1)
**ESRD**						
Unadjusted	53.8 (50.1 to 57.5)	11.5 (9.2 to 13.9)	15.5 (12.8 to 18.2)	3.2 (1.9 to 4.5)	3.1 (1.8 to 4.4)	12.9 (10.4 to 15.4)
Age/sex adjusted	53.0 (42.3 to 63.6)	12.0 (8.6 to 15.4)	15.7 (11.6 to 19.8)	3.1 (-15.3 to 21.6)	3.1 (1.7 to 4.6)	13.1 (9.5 to 16.6)
**Total (cases)**	10201	5026	3285	1888	683	4,133

CVD, cardiovascular disease; eGFR, estimated glomerular filtration rate; ESRD, end-stage renal disease.

### Sensitivity and sex-specific analyses

After excluding 36,610 patients with creatinine measures obtained during the last 90 days prior to death, proportions were similar to the main analysis (S3–S6 Tables).

We also explored possible sex-differences in causes of death (S7–S10 Tables). Briefly, at study inclusion, there were more women than men, and men had a higher prevalence of diabetes (21% vs 15%, in women). In both sexes, CVD was the main cause of death with lower eGFR (S8 Table), although ischemic heart disease was more often reported as a cause of death in men than in women at all eGFR strata (46% vs 36% overall in men versus women, S9 Table). Death due to neurologic diseases, including dementia, more often affected women than men in all eGFR categories (35% vs 26% in eGFR>90, and 39% vs 25% in eGFR 60–89 ml/min/1.73 m^2^). (S10 Table). Diabetes, on the other hand, was more often the cause of death in men compared to women (for instance, 10.5% vs 6.0% in eGFR 45–59 ml/min and 32.5% vs 21.2% in ESRD, S10 Table). Deaths due to accidents and suicides were more common in men than women (in total, 19% vs 11%)–a difference that persisted across all eGFR strata.

## Discussion

In this region-representative population-based Swedish healthcare extraction, we observe that the pattern of causes of death differs across CKD stages. Our findings lend further validity to recent reports [3, 4, 6, 7] and expand these observations to Northern Europe. Besides confirming previous reports, we provide new information by including individuals with ESRD as a separate category and by providing a sex-specific analysis of causes of death.

An important observation in our study is the gradual increase in the age-sex-adjusted proportion of CVD deaths with lower eGFR strata, a shift that becomes apparent already in the category of eGFR<60–45 ml/min/1.73 m^2^. This and preceding evidence [3, 4, 6, 7] illustrate collectively the burden that CVD imposes in individuals with CKD. A recent estimation from the 2013 Global Burden of Disease (GBD) consortium, suggested that reduced GFR was associated with 4% of deaths worldwide, and more than half of these attributable deaths were CVD related [10]. While numerous studies have demonstrated a consistent association between reduced GFR and specific CV events [11–13], our analysis expands this evidence by exploring the differential distribution of these across eGFR strata.

Ischemic heart disease and cerebrovascular disease were the most common causes of CVD death overall, but we did not find striking differences in their proportion across worse eGFR strata. In contrast, deaths attributed to heart failure, arrhythmias and valvular disease tended to rise with lower eGFR. This observation is useful from a clinical perspective as different cardiovascular events require different interventions. From a mechanistic perspective, these observations support the hypothesis that other factors than atherosclerosis, such as vascular calcification and chronic volume overload, may be more intrinsically linked to increased cardiac workload at lower eGFR. Various mechanisms have been proposed, such as osteoblast-like cells formation, excess calciprotein particles and metabolic insults of calcium-phosphate retention, diabetes, dyslipidemia, or oxidative stress, in the vessel wall [14–16].

Cancer was the main cause of death in individuals with eGFR>90 ml/min/1.73 m^2^ in our study, but progressively moved to second and third place with lower eGFR. Again, these proportion estimates again agree with those of other countries [3, 4, 6, 7] and may be explained by other complications (namely cardiovascular diseases and infections) superseding the likelihood of dying from cancer, which occurs more commonly at older ages. We note that in prospective analyses, the association between eGFR and cancer death is, at most, weak and inconclusive [6, 17, 18], possibly because of insufficient control of confounders in registry-based analyses. One UK study that accounted for important confounders such as blood pressure and lipids could not observe any significant association of CKD, defined as reduced eGFR or elevated proteinuria, with cancer mortality [19]. Additionally, it is also possible that cancer incidence (notably renal and urothelial cancers) [20] increases with lower eGFR, but that such patients die of other causes.

Deaths due to infection increased in patients with eGFR<60 ml/min/1.73 m^2^, and remained similarly elevated with lower eGFR categories. This agrees with population-based studies suggesting that the incidence of community-acquired infections overall and of those severe enough to require hospitalization are generally increased with lower eGFR, and are accounted for primarily by pneumonia, urinary tract infections, sepsis/bacteremia and cystitis [21–24]. It has been proposed that there is a biologic plausibility for the causality in these associations given the well-known effect of uremic toxicity on T lymphocyte and antigen-presenting cells [25] and the generation of oxidative stress [26], factors that alter both cellular and humoral immunity. Further, this may also be generally explained by excess comorbidity and frailty associated with CKD, which increase both the susceptibility to illness and the risk of subsequent death.

A novelty in our study is the performance of sex-specific analyses, in view of the insufficiently understood gender differences in CKD epidemics and outcomes [27, 28].Although sex-specific causes of death agree in general with our main analysis, there are some intriguing differences. For instance, death due to ischemic heart disease seems more common among men throughout all CKD strata. This agrees with the general population knowledge that death from coronary heart disease is more common in men than in women, although the gap between sexes is thought to narrow after menopause [29]. Also in agreement with previous knowledge, cancer, accidents and diabetes were more common among men [30]. Conversely, deaths attributed to neurologic diseases and dementias were higher in women, an issue possibly explained by the well-described longer life expectancy of women in the general population.

In the interpretation of our findings, we remind the reader that in this study we investigate pattern of causes of deaths, not mortality rates. It is indeed well-described that all-causes of death, both cardiovascular and non-cardiovascular, increase with lower eGFR [3–6]. For instance, in the region of Stockholm where our study takes place, Neovius et al. reported a hazard ratio of 4.1 and 3.3 for CVD and non-CVD mortality, respectively, in patients with CKD stages IV-V compared to persons with normal kidney function [31]. Our analysis, however, addresses a complementary question: although mortality risk may be increased, the event can be less frequent/common in proportion, which can be useful to inform patients and physicians of likely risks. For instance, although cancer-related mortality is increased with lower eGFR [32], we observe that their relative proportion is, however, less common, as other causes of death override.

The reader should also bear in mind some study strengths, such as the large and region-representative coverage of the sampling, with universal healthcare (which minimizes healthcare access bias) and government centralized outcome ascertainment with virtually no losses to follow up. Nevertheless, we have other limitations, mainly related to the inherent biases of using ICD reporting in administrative databases for causes of death [33], especially in comorbid and complex patients such as the elderly and those with advanced CKD. Even so, because of strong similarities with reports from other countries, we are confident of the potential generalizability of our findings. We used single measurement of eGFR, which may lead to misclassification of exposure. For that reason, in part, our analysis attempted to select the closest ambulatory eGFR estimation prior to death, which may more representative of their true kidney function when the event occurred. Further, we did a sensitivity analysis that excluded all eGFR measurements within three months before death and results were like those in the primary analysis. Unfortunately, we could not assess the associations with albuminuria because this metric is less often investigated in healthcare in our region and therefore would impose a strong selection bias.

To conclude, in this region-representative Swedish healthcare extraction, we report differences regarding causes of death across different CKD stages. Because prevention and management can be substantially different across diseases, our study may have implications for both clinical management and public health. Although CKD has been associated with an increased risk of many adverse health outcomes, characterizing the relative distribution of cause-specific deaths by eGFR category provides a comprehensive description of the cause-specific burden of disease in CKD. This information may be helpful for prioritizing resource allocation to future interventions, and inform decisions regarding patient management, emphasis on prevention strategies, and health service planning. For instance, deaths attributed to genitourinary diseases rise, perhaps not unexpectedly, with lower eGFR in our study, something that could be explained by reverse causation. However, it is still an important observation, given that most individuals with CKD in the greater community are unaware of their kidney dysfunction, and may not even be diagnosed [1], and this information can be used to justify more thorough patient investigations if in suspicion of disease.

## Supporting information

S1 Table Classification of causes of death according to ICD-10 codes. ICD, International Classification of Diseases, 10^th^ revision.
(DOCX)

S2 Table eGFR strata, by sex. (DOCX)

S3 Table Sensitivity analysis. Participant characteristics after exclusion of creatinine measurments from the last 90 days of life. CVD, cardiovascular disease, eGFR, estimated glomerular filtration rate, ESRD, end-stage renal disease.
(DOCX)

S4 Table Sensitivity analysis. Causes of death after exclusion of creatinine measures from the last 90 days of life. CVD, cardiovascular disease, eGFR, estimated glomerular filtration rate, ESRD, end-stage renal disease
(DOCX)

S5 Table Sensitivity analysis. Other causes of death after exclusion of creatinine measures from the last 90 days of life.
(DOCX)

S6 Table Sensitivity analysis. CVD causes of death after exclusion of creatinine measures from the last 90 days of life.
(DOCX)

S7 Table Female and male participant characteristics. CVD, cardiovascular disease.
(DOCX)

S8 Table Age-adjusted proportions (in %) in causes of death for men and women separately. CVD, cardiovascular disease, ESRD, end stage renal disease.
(DOCX)

S9 Table Age adjusted proportions (in %) of cardiovascular causes of death for men and women separately. CVD, cardiovascular disease.
(DOCX)

S10 Table Age adjusted proportions (in %) of other causes of death for men and women separately. Misc., miscellaneous. ESRD, end stage renal disease.
(DOCX)
