# Supplementary material for: Causes of death across categories of estimated glomerular filtration rate: The Stockholm CREAtinine Measurements (SCREAM) project
Source: PLoS One. 2019 Jan 16;14(1):e0209440. doi: 10.1371/journal.pone.0209440 (PMC6334920; doi:10.1371/journal.pone.0209440)
Supplement: S1 Table — ICD, International Classification of Diseases, 10th revision. (DOCX) [file pone.0209440.s001.docx]

| CARDIOVASCULAR DISEASE | |
| --- | --- |
| ischaemic heart disease | I20 to I25 |
| heart failure | I11, I13, I25.5, I42.0, I42.5 to 9, I43, I50 |
| cerebrovascular disease | G45, G46, H34.0, I 60 to I69 |
| arrythmia | I45 to I49, R00, Z45, Z95 |
| Valvular heart disease  other | I05 to I10, I34 to I37  I26, I51, I70 to I79, T82 |
| infection |  |
|  | A01 to B99, K35 to K38, K80 to K82, I30.1, I32.0, I32.1, I33, I38.9, I39.8, I40 to 41, N11, N39, G00 to G09, J1, J2, J44.0, J85, J96.8A |
| Cancer |  |
|  | C00 to C97, D46 D1 to D3 |
| other |  |
| Chronic lung disease  DIABETIC COMPLICATIONS  NEUROLOGY AND DEMENTIA  GASTROINTESTINAL  Accidents and suicide  Other | J4 to J67, J84.1, J96.1  E10 to E15  F00 to F04, G03.0 to G03.8, G21 to G22, G3, G99  K00 to K93 excluding K35 to K38, K80 to K82  V to Y  All other codes |
|  |  |
