# Supplementary material for: Causes of death across categories of estimated glomerular filtration rate: The Stockholm CREAtinine Measurements (SCREAM) project
Source: PLoS One. 2019 Jan 16;14(1):e0209440. doi: 10.1371/journal.pone.0209440 (PMC6334920; doi:10.1371/journal.pone.0209440)
Supplement: S2 Table — (DOCX) [file pone.0209440.s002.docx]

| **eGFR strata, ml/min /1.73 m^2^** | Female | Male |
| --- | --- | --- |
| >90, N (%) | 4,926 (13.4) | 7,612 (22.6) |
| 60- 89, N (%) | 15,701 (42.6) | 13,236 (39.3) |
| 45-59, N (%) | 6,988 (19) | 5,272 (15.7) |
| 30-44, N (%) | 5,645 (15.3) | 4,223 (12.5) |
| 15-29, N (%) | 2,879 (7.8) | 2,350 (7) |
| ESRD | 739 (2) | 976 (2.9) |
| Total | 36,878 | 33,669 |
