# Supplementary material for: Causes of death across categories of estimated glomerular filtration rate: The Stockholm CREAtinine Measurements (SCREAM) project
Source: PLoS One. 2019 Jan 16;14(1):e0209440. doi: 10.1371/journal.pone.0209440 (PMC6334920; doi:10.1371/journal.pone.0209440)
Supplement: S3 Table — Participant characteristics after exclusion of creatinine measurments from the last 90 days of life. CVD, cardiovascular disease, eGFR, estimated glomerular filtration rate, ESRD, end-stage renal disease. (DOCX) [file pone.0209440.s003.docx]

| **Participant Characteristics** | **Overall** | **Death attributed to** | | | |
| --- | --- | --- | --- | --- | --- |
|  |  | **CVD** | **Cancer** | **Infection** | **Other** |
| N (100%) | 33937 (100) | 12741 (38) | 8788 (26) | 1620 (4.8) | 10788 (32) |
| Women, N (%) | 18484 (54.5) | 7087 (55.6) | 4461 (50.8) | 841 (51.9) | 6095 (56.5) |
| Diabetes, N (%) | 5849 (17.2) | 2307 (18.1) | 1346 (15.3) | 320 (19.8) | 1876 (17.4) |
| CVD, N (%) | 14286 (42.1) | 7085 (55.6) | 2367 (26.9) | 795 (49.1) | 4039 (37.4) |
| **eGFR (ml/min/1.73^2^)** | | | | | |
| >90, N (%) | 5837 (17.2) | 1223 (9.6) | 2373 (27.0) | 202 (12.5) | 2039 (18.9) |
| 60 to 89, N (%) | 15235 (44.9) | 5620 (44.1) | 4026 (45.8) | 702 (43.3) | 4887 (45.3) |
| 45 to 59, N (%) | 6236 (18.4) | 2758 (21.6) | 1301 (14.8) | 319 (19.7) | 1858 (17.2) |
| 30 to 44, N (%) | 4560 (13.4) | 2187 (17.2) | 751 (8.5) | 283 (17.5) | 1339 (12.4) |
| 15 to 29, N (%) | 1721 (5.1) | 824 (6.5) | 282 (3.2) | 100 (6.2) | 515 (4.8) |
| ESRD | 348 (1.0) | 129 (1.0) | 55 (0.6) | 14 (0.9) | 150 (1.4) |
