# Supplementary material for: Causes of death across categories of estimated glomerular filtration rate: The Stockholm CREAtinine Measurements (SCREAM) project
Source: PLoS One. 2019 Jan 16;14(1):e0209440. doi: 10.1371/journal.pone.0209440 (PMC6334920; doi:10.1371/journal.pone.0209440)
Supplement: S4 Table — Causes of death after exclusion of creatinine measures from the last 90 days of life. CVD, cardiovascular disease, eGFR, estimated glomerular filtration rate, ESRD, end-stage renal disease (DOCX) [file pone.0209440.s004.docx]

| **eGFR strata** | **CVD** | **Cancer** | **Infection** | **Other** |
| --- | --- | --- | --- | --- |
| **>90 ml/min/1.73 m^2^** | | | | |
| Unadjusted | 21.0 (19.9-22.0) | 40.7 (39.4-41.9) | 3.46 (3.0-3.93) | 34.9 (33.7-36.2) |
| Age/sex adjusted | 24.9 (23.6-26.2) | 36.1 (34.7-37.6) | 3.8 (3.2-4.4) | 35.1 (33.7-36.5) |
| **60 to 89 ml/min/1.73 m^2^** | | | | |
| Unadjusted | 36.9 (36.1-37.7) | 26.4 (25.7-27.1) | 4.6 (4.3-4.9) | 32.1 (31.3-32.8) |
| Age/sex adjusted | 35.8 (35.1-36.6) | 27.1 (26.4-27.8) | 4.6 (4.2-4.9) | 32.5 (31.7-33.2) |
| **45 to 59 ml/min/1.73 m^2^** | | | | |
| Unadjusted | 44.2 (43.0-45.5) | 20.9 (19.9-21.9) | 5.1 (4.6-5.7) | 29.8 (28.7-30.9) |
| Age/sex adjusted | 42.7 (41.4-43.9) | 22.0 (20.9-23.0) | 5.1 (4.5-5.7) | 30.2 (29.1-31.4) |
| **30 to 44 ml/min/1.73 m^2^** | | | | |
| Unadjusted | 48.0 (46.-49.4) | 16.5 (15.4-17.5) | 6.2 (5.5-6.9) | 29.4 (28.0-30.7) |
| Age/sex adjusted | 46.3 (44.9-47.8) | 17.5 (16.4-18.7) | 6.2 (5.5-6.9) | 30.0(28.6-31.3) |
| **15 to 29 ml/min/1.73 m^2^** | | | | |
| Unadjusted | 47.9 (45.5-50.2) | 16.4 (14,6-18.1) | 5.8 (4.7-6.9) | 29.9 (27.8-32.1) |
| Age/sex adjusted | 46.4 (44.0-48.7) | 17.3 (15.4-19.1) | 5.8 (4.7-6.9) | 30.6 (28.4-32.8) |
| **ESRD** | | | | |
| Unadjusted | 37.1 (32.0-42.1) | 15.8 (12.0-19.6) | 4.0 (2.0-6.1) | 43.1 (37.9-48.3) |
| Age/sex adjusted | 36.1 (31.0-41.2) | 15.8 (12.0-19.7) | 3.9 (1.9-5.9) | 44.1 (38.9-49.4) |
