# Supplementary material for: Causes of death across categories of estimated glomerular filtration rate: The Stockholm CREAtinine Measurements (SCREAM) project
Source: PLoS One. 2019 Jan 16;14(1):e0209440. doi: 10.1371/journal.pone.0209440 (PMC6334920; doi:10.1371/journal.pone.0209440)
Supplement: S5 Table — Other causes of death after exclusion of creatinine measures from the last 90 days of life. (DOCX) [file pone.0209440.s005.docx]

| eGFR strata | Chronic Lung Disease | Accidents and suicide | Neurology incl. dementia | Gastrointestinal Tract | Diabetes | Genitourinary Disease | Miscellaneous |
| --- | --- | --- | --- | --- | --- | --- | --- |
| Total | 1437 | 1315 | 2416 | 1135 | 773 | 481 | 1795 |
| >90 ml/min/1.73 m^2^ | | | | | | | |
| Unadjusted | 14.7 (13.2-16.3) | 28.7 (26.8-30.7) | 21.4 (19.6-23.2) | 14.8 (13.3-16.4) | 4.8 (3.8-5.7) | 0,44 (0,15-0,72) | 15.2 (13.6-16.7) |
| Age/sex adjusted | 19.9 (17.8-22.1) | 14.7 (12.9-16.4) | 33.0 (30.4-35.6) | 12.0 (10.3-13.6) | 4,4 (3.4-5.4) | 0.54 (0.18-0.91) | 15.5 (13.6-17.4) |
| 60 to 89 ml/min/1.73 m^2^ | | | | | | | |
| Unadjusted | 14.7 (13.7-15.6) | 13.2 (12.3-14.2) | 38.4 (37.1-39.8) | 8.63 (7.84-9.4) | 4.05 (3.5-4.6) | 0.92 (0.66-1.19) | 20.1 (19.0-21.2) |
| Age/sex adjusted | 14.0 (13.0-15.1) | 14.7 (13.7-15.8) | 35.2 (33.7-36.6) | 9.22 (8.37-10.1) | 4.31 (3.71-4.91) | 0.82 (0.57-1.08) | 21.7 (20.5-22.9) |
| 45 to 59 ml/min/1.73 m^2^ | | | | | | | |
| Unadjusted | 15.5 (13.9-17.1) | 11.9 (10.4-13.4) | 33.6 (31.4-35.7) | 8.2 (6.9-9.4) | 6.19 (5.1-7.29) | 2.2 (1.54-2.87) | 22.4 (20.5-24.3) |
| Age/sex adjusted | 14.5 (12.9-16.1) | 14.2 (12.5-15.9) | 29.5 (27.4-31.6) | 9.0 (7.6-10.4) | 6.7 (5.5-7.9) | 1.97 (1.32-2.61) | 24.0 (22.0-26.0) |
| 30 to 44 ml/min/1.73 m^2^ | | | | | | | |
| Unadjusted | 14.5 (12.6-16.4) | 12.9 (11.1-14.7) | 28.5 (26.1-30.9) | 9.1 (7.6-10.7) | 10.3 (8.7-12.0) | 3.5 (2.51-4.58) | 20.8 (18.6-23.0) |
| Age/sex adjusted | 13.4 (11.5-15.2) | 15.5 (13.4-17.6) | 24.5 (22.2-26.7) | 10.1 (8.4-11.8) | 11.2 (9.4-13.0) | 3.1 (2.20-4.12) | 22.0 (19.7-24.3) |
| 15 to 29 ml/min/1.73 m^2^ | | | | | | | |
| Unadjusted | 10.4 (7.8-13.1) | 13.3 (10.4-16.3) | 18.8 (15.4-22.2) | 11.2 (8.5-13.9) | 10.2 (7.6-12.9) | 11.2 (8.5-13.9) | 24.4 (20.7-28.1) |
| Age/sex adjusted | 9.5 (7.1-12.0) | 15.6 (12.2-18.9) | 16.2 (13.1-19.3) | 12.1 (9.2-15.1) | 10.8 (8.1-13.6) | 9.7 (7.0-12.3) | 25.7 (21.8-29.6) |
| ESRD | | | | | | | |
| Unadjusted | 2.6 (0.08-5.24) | 1.33 (-0.50-3.16) | 6.66 (2.67-10.65) | 4.0 (0.86-7.13) | 30.6 (23.2-38.04) | 38.6 (30.8-46.4) | 16.0 (10.1-21.8) |
| Age/sex adjusted | 2.7 (0.08-5.33) | 1.35 (-0.52-3.23) | 6.72 (2.66-10.78) | 4.26 (0.916-7.61) | 32.4 (24.6-40.2) | 34.6 (26.6-42.5) | 17.8 (11.3-24.3) |
