# Supplementary material for: Causes of death across categories of estimated glomerular filtration rate: The Stockholm CREAtinine Measurements (SCREAM) project
Source: PLoS One. 2019 Jan 16;14(1):e0209440. doi: 10.1371/journal.pone.0209440 (PMC6334920; doi:10.1371/journal.pone.0209440)
Supplement: S6 Table — CVD causes of death after exclusion of creatinine measures from the last 90 days of life. (DOCX) [file pone.0209440.s006.docx]

| **eGFR strata** | **Ischemic Heart Disease** | **Cerebrovascular disease** | **Heart Failure** | **Arrhythmia** | **Valvular Disease** | **Other CVD** |
| --- | --- | --- | --- | --- | --- | --- |
| Total | 5181 | 2270 | 1680 | 964 | 371 | 2009 |
| **>90 ml/min/1.73 m^2^** | | | | | | |
| Unadjusted | 48.5 (45.7-51.3) | 22.4 (20.0-24.7) | 5.6 (4.34-6.93) | 1.88 (1.11-2.64) | 1.06 (0.48-1.63) | 20.4 (18.1-22.7) |
| Age/sex adjusted | 42.7 (38.1-47.4) | 26.9 (23.2-30.5) | 8.20 (6.21-10.19) | 2.58 (-5.28-10.4) | 1.06 (0.43-1.70) | 18.4 (15.5-21.3) |
| **60 to 89 ml/min/1.73 m^2^** | | | | | | |
| Unadjusted | 37.3 (36.0-38.6) | 24.0 (22.9-25.2) | 12.0 (11.1-12.8) | 7.1 (6.46-7.80) | 2.02 (1.65-2.39) | 17.3 (16.3-18.3) |
| Age/sex adjusted | 37.9 (30.0-45.7) | 24.3 (19.2-29.4) | 11.2 (8.80-13.7) | 6.58 (-12.5-25.6) | 2.07 (1.50-2.63) | 17.8 (14.0-21.6) |
| **45 to 59 ml/min/1.73 m^2^** | | | | | | |
| Unadjusted | 38.2 (36.4-40.0) | 21.2 (19.7-22.8) | 13.0 (11.7-14.3) | 8.8 (7.82-9.94) | 2.93 (2.30-3.56) | 15.5 (14.2-16.9) |
| Age/sex adjusted | 40.0 (29.9-50.0) | 21.0 (15.6-26.4) | 11.7 (8.62-14.9) | 7.9 (-14.7-30.6) | 2.9 (1.99-3.95) | 16.1 (11.9-20.4) |
| **30 to 44 ml/min/1.73 m^2^** | | | | | | |
| Unadjusted | 38.4 (36.4-40.4) | 18.8(17.1-20.4) | 15.4 (13.9-16.9) | 8.82 (7.63-10.0) | 3.47(2.70-4.24) | 14.9 (13.4-16.4) |
| Age/sex adjusted | 40.3 (30.2-50.4) | 18.6 (13.8-23.5) | 13.9 (10.2-17.6) | 7.8 (-14.6-30.4) | 3.54 (2.36-4.71) | 15.5 (11.4-19.7) |
| **15 to 29 ml/min/1.73 m^2^** | | | | | | |
| Unadjusted | 43.8 (40.4-47.1) | 13.8 (11.4-16.1) | 17.4 (14.8-20.0) | 6.9 (5.18-8.65) | 3.15 (1.96-4.34) | 14.8 (12.3-17.2) |
| Age/sex adjusted | 45.5 (36.0-54.9) | 13.7 (10.2-17.3) | 15.9 (11.9-19.8) | 6.24 (-11.9-24.4) | 3.2 (1.84-4.57) | 15.3 (11.4-19.2) |
| **ESRD** | | | | | | |
| Unadjusted | 54.2 (45.6-62.8) | 11.6 (6.09-17.1) | 14.7 (8.61-20.84) | 3.875 (0.54-7.20) | 1.55 (-0.58-3.68) | 13.95 (7.97-19.93) |
| Age/sex adjusted | 53.6 (42.9-64.3) | 12.0 (6.19-17.9) | 14.5 (8.21-20.8) | 3.72 (-7.85-15.30) | 1.61 (-0.61-3.84) | 14.38 (8.01-20.7) |
