# Supplementary material for: Causes of death across categories of estimated glomerular filtration rate: The Stockholm CREAtinine Measurements (SCREAM) project
Source: PLoS One. 2019 Jan 16;14(1):e0209440. doi: 10.1371/journal.pone.0209440 (PMC6334920; doi:10.1371/journal.pone.0209440)
Supplement: S7 Table — CVD, cardiovascular disease. (DOCX) [file pone.0209440.s007.docx]

|  |  |  | **Death attributed to** | | | |
| --- | --- | --- | --- | --- | --- | --- |
| **Participant characteristics** | **Sex** | Overall | **CVD** | **Cancer** | **Infection** | **Other** |
| N (%) | Women | 36,878 (100) | 13,573 (36.8) | 10,649 (28.9) | 1,612 (4.3) | 11,044 (30) |
|  | Men | 33,669 (100) | 11,643 (34.6) | 11,292 (33.5) | 1,638 (4.9) | 9,096 (27.0) |
| Age, years (median, 10^th^-90^th^ p) | Women | 85 (64 to 94) | 88 (75 to 96) | 75 (56 to 89) | 87 (70 to 95) | 86 (67 to 95) |
|  | Men | 80 (60 to 91) | 83 (65 to 92) | 75 (58 to 88) | 84 (63 to 93) | 80 (56 to 91) |
| Diabetes, N (%) | Women | 15.3 | 16.4 | 12.9 | 18.2 | 15.9 |
|  | Men | 21.4 | 23.4 | 18.0 | 21.1 | 23.1 |
| CVD, N (%) | Women | 42.5 | 59.3 | 23.2 | 49.0 | 39.4 |
|  | Men | 46.7 | 63.2 | 31.1 | 52.3 | 43.9 |
