# Supplementary material for: Causes of death across categories of estimated glomerular filtration rate: The Stockholm CREAtinine Measurements (SCREAM) project
Source: PLoS One. 2019 Jan 16;14(1):e0209440. doi: 10.1371/journal.pone.0209440 (PMC6334920; doi:10.1371/journal.pone.0209440)
Supplement: S8 Table — CVD, cardiovascular disease, ESRD, end stage renal disease. (DOCX) [file pone.0209440.s008.docx]

|  |  | **Death attributed to** | | | |
| --- | --- | --- | --- | --- | --- |
| **eGFR** | **Sex** | **CVD** | **Cancer** | **Infection** | **Other** |
| >90 | Male | 22.6 (21.5 to 23.6) | 45.9 (44.6 to 47.2) | 3.8 (3.4 to 4.3) | 27.7 (26.6 to 28.8) |
|  | Female | 18.1 (16.8 to 19.4) | 49.6 (48.0 to 51.3) | 3.2 (2.6 to 3.8) | 29.1 (27.6 to 30.6) |
| 60 to 89 | Male | 33.5 (32.6 to 34.3) | 34.9 (34.0 to 35.7) | 4.7 (4.3 to 5.1) | 27.0 (26.2 to 27.7) |
|  | Female | 33.6 (32.8 to 34.3) | 30.6 (29.8 to 31.3) | 3.9 (3.6 to 4.2) | 31.9 (31.2 to 32.7) |
| 45 to 59 | Male | 39.5 (38.2 to 40.8) | 29.2 (27.9 to 30.4) | 5.5 (4.9 to 6.2) | 25.8 (24.6 to 27.0) |
|  | Female | 41.6 (40.4 to 42.8) | 23.5 (22.5 to 24.5) | 5.1 (4.6 to 5.6) | 29.8 (28.7 to 30.9) |
| 30 to 44 | Male | 44.2 (42.7 to 45.8) | 24.9 (23.6 to 26.2) | 5.8 (5.1 to 6.5) | 25.1 (23.7 to 26.4) |
|  | Female | 44.9 (43.6 to 46.3) | 19.0 (17.9 to 20.1) | 6.0 (5.4 to 6.6) | 30.1 (28.8 to 31.3) |
| 15 to 29 | Male | 44.0 (42.0 to 46.0) | 22.2 (20.5 to 23.9) | 5.9 (4.9 to 6.9) | 27.9 (26.0 to 29.7) |
|  | Female | 47.2 (45.3 to 49.0) | 17.2 (15.8 to 18.7) | 5.4 (4.5 to 6.2) | 30.2 (28.5 to 31.9) |
| ESRD | Male | 38.4 (35.3 to 41.4) | 15.4 (13.1 to 17.6) | 5.4 (4.0 to 6.8) | 40.9 (37.8 to 44.0) |
|  | Female | 39.6 (36.1 to 43.2) | 12.7 (10.3 to 15.2) | 4.7 (3.1 to 6.2) | 43.0 (39.4 to 46.5) |
| **Total (N)** | Male | 11643 | 11292 | 1638 | 9096 |
|  | Female | 13573 | 10649 | 1612 | 11044 |
| **Total (%)** | Male | 34.6 | 33.5 | 4.9 | 16.1 |
|  | Female | 36.8 | 28.9 | 4.3 | 13.5 |
