# Supplementary material for: Causes of death across categories of estimated glomerular filtration rate: The Stockholm CREAtinine Measurements (SCREAM) project
Source: PLoS One. 2019 Jan 16;14(1):e0209440. doi: 10.1371/journal.pone.0209440 (PMC6334920; doi:10.1371/journal.pone.0209440)
Supplement: S9 Table — CVD, cardiovascular disease. (DOCX) [file pone.0209440.s009.docx]

|  |  | **Death attributed to** | | | | | |
| --- | --- | --- | --- | --- | --- | --- | --- |
| **eGFR strata** | **Sex** | **Ischaemic Heart Disease** | **Cerebrovascular disease** | **Heart Failure** | **Arrythmia** | **Valvular disease** | **Other CVD** |
| >**90 ml/min/1.73 m^2^** | Male | 47.1 (39.8-54.5) | 25.2 (20.8-29.6) | 8.1 (6.1-10.0) | 2.7 (-11.4-16.8) | 0.9 (0.4-1.3) | 16.1 (13.0-19.1) |
|  | Female | 37.4 (27.1-47.7) | 28.5 (20.3-36.6) | 8.9 (-11.5-23.2) | 2.6 (-5.9-11.1) | 1.5 (-6.8-9.8) | 21.1 (14.8-27.5) |
| **60 to 89 ml/min/1.73 m^2^** | Male | 43.8 (29.2-58.3) | 21.2 (14.1-28.4) | 9.7 (6.4-13.0) | 6.2 (-24.8-37.2) | 2.2 (1.4-3.1) | 16.9 (11.2-22.6) |
|  | Female | 33.8 (19.4-48.2) | 25.3 (14.5-36.2) | 12.6 (-15.1-40.3) | 7.7 (-15.9-31.3) | 2.4 (-10.5-15.2) | 18.2 (10.4-26.0) |
| **45 to 59 ml/min/1.73 m^2^** | Male | 47.6 (28.1-67.0) | 16.2 (9.4-23.0) | 10.4 (6.0-14.9) | 7.6 (-30.0-45.2) | 2.8 (1.5-4.2) | 15.4 (8.9-21.8) |
|  | Female | 35.6 (18.0-53.2) | 22.0 (11.1-33.0) | 13.8 (-16.3-43.9) | 9.4 (-19.0-37.8) | 3.0 (-13.3-19.3) | 16.2 (8.1-24.3) |
| **30 to 44 ml/min/1.73 m^2^** | Male | 47.1 (30.0-64.4) | 15.6 (9.6-21.5) | 11.1 (6.8-15.4) | 6.9 (-27.3-41.0) | 4.2 (2.4-6.0) | 15.2 (9.4-21.0) |
|  | Female | 37.1 (17.0-57.1) | 17.5 (8.0-27.1) | 16.4 (-18.3-51.3) | 8.6 (-17.6-34.7) | 3.7 (-16.2-23.6) | 16.7 (5.8-23.3) |
| **15 to 29 ml/min/1.73 m^2^** | Male | 52.2 (38.1-63.3) | 10.0 (6.8-13.2) | 15.1 (10.6-19.6) | 4.9 (-20.1-30.0) | 2.5 (1.4-3.7) | 15.2 (10.6-19.8) |
|  | Female | 40.1 (16.8-65.1) | 13.5 (5.4-21.5) | 18.9 (-19.9-57.7) | 8.1 (-16.9-33.1) | 4.0 (-17.5-25-5) | 14.6 (5.9-22.9) |
| **ESRD** | Male | 58.0 (49.6-66.4) | 13.3 (9.5-17.0) | 12.2 (8.6-15.8) | 2.2 (-9.3-13.7) | 2.6 (1.0-4.2) | 11.7 (8.2-15.3) |
|  | Female | 49.6 (23.2-76.0) | 9.8 (3.7-16.0) | 19.2 (-19.9-58.7) | 4.3 (-9.6-18.1) | 2.7 (-12.0-17.2) | 14.4 (10.4-18.2) |
| **Total (N)** | Male | 5353 | 2127 | 1284 | 766 | 285 | 1828 |
|  | Female | 4848 | 2899 | 2001 | 1122 | 398 | 2305 |
| **Total (%)** | Male | 46.0 | 18.3 | 11.0 | 6.6 | 2.45 | 15.7 |
|  | Female | 35.7 | 21.4 | 14.7 | 8.3 | 2.9 | 17.0 |
