# Supplementary material for: Causes of death across categories of estimated glomerular filtration rate: The Stockholm CREAtinine Measurements (SCREAM) project
Source: PLoS One. 2019 Jan 16;14(1):e0209440. doi: 10.1371/journal.pone.0209440 (PMC6334920; doi:10.1371/journal.pone.0209440)
Supplement: S10 Table — Misc., miscellaneous. ESRD, end stage renal disease. (DOCX) [file pone.0209440.s010.docx]

|  |  | **Death attributed to** | | | | | | |
| --- | --- | --- | --- | --- | --- | --- | --- | --- |
| **eGFR** | **Sex** | **Chronic Lung Disease** | **Accidents and suicide** | **Neurology incl. dementia** | **Gastrointestinal Tract** | **Diabetes** | **Gen urin dis.** | **Misc.** |
| >90 ml/min/1.73 m^2^ | Male | 18.8 (16.8-20.8) | 21.0 (18.7-22.6) | 25.8 (23.6-28.1) | 14.3 (12.6-16.0) | 4.6 (3.7-5.6) | 0.7 (0.3-1.1) | 15.0 (13.3-16.7) |
|  | Female | 24.0 (21.1-26.9) | 11.8 (9.8-13.8) | 34.9 (31.6-38.2) | 10.3 (8.4-12.2) | 3.8 (2.6-5.0) | 0.19 (-0.08-0.05) | 15.0 (12.8-17.3) |
| 60 to 89 ml/min/1.73 m^2^ | Male | 15.6 (14.3-16.9) | 20.1 (18.1-26.5) | 25.3 (23.8-26.9) | 12.4 (11.2-13.6) | 6.3 (5.4-7.1) | 1.5 (1.1-1.9) | 18.8 (17.4-20.2) |
|  | Female | 14.4 (13.4-15.4) | 10.9 (10.0-11.8) | 38.8 (37.4-0.2) | 8.4 (7.6-9.2) | 3.8 (3.3-3.4) | 0.76 (0.51-0.10) | 22.9 (21.7-24.1) |
| 45 to 59 ml/min/1.73 m^2^ | Male | 14.6 (12.7-16.5) | 16.4 (14.2-18.4) | 22.5 (20.2-24.7) | 12.5 (10.6-14.4) | 10.5 (8.8-12.3) | 3.0 (2.1-4.0) | 20.4 (18.1-22.7) |
|  | Female | 15.3 (13.8-16.9) | 11.6 (10.2-13.1) | 30.1 (29.0-33.0) | 10.2 (8.8-11.5) | 6.0 (5.0-7.1) | 1.5 (0.98-2.0) | 24.3 (22.4-26.2) |
| 30 to 44 ml/min/1.73 m^2^ | Male | 13.5 (11.4-15.5) | 17.9 (15.3-20.5) | 17.3 (15.1-19.6) | 12.6 (10.4-14.7) | 13.6 (11.4-15.9) | 5.5 (4.1-7.0) | 19.5 (17.0-22.1) |
|  | Female | 13.8 (12.1-15.1) | 11.6 (10.0-13.2) | 27.8 (25.6-29.9) | 11.0 (9.5-12.6) | 8.8 (7.4-10.2) | 2.9 (2.1-3.7) | 24.1 (22.0-26.1) |
| 15 to 29 ml/min/1.73 m^2^ | Male | 8.4 (6.4-10.5) | 14.5 (11.5-17.4) | 12.9 (10.4-15.3) | 14.3 (11.4-17.1) | 16.0 (13.0-19.0) | 13.8 (10.9-16.6) | 20.2 (16.9-23.4) |
|  | Female | 10.7 (8.6-12.7) | 10.1 (8.0-12.2) | 21.7 (19.0-24.4) | 11.9 (9.7-14.1) | 11.2 (9.1-13.4) | 9.8 (7.8-11.8) | 24.6 (21.6-27.5) |
| ESRD | Male | 3.5 (1.7-5.2) | 4.9 (2.6-7.1) | 6.8 (4.4-9.2) | 4.9 (2.7-7.1) | 32.5 (27.6-37.4) | 34.0 (28.9-39.0) | 13.5 (10.0-17.1) |
|  | Female | 4.2 (2.0-6.4) | 1.4 (0.17-2.6) | 11.8 (8.2-15.3) | 4.9 (2.5-7.3) | 21.1 (16.6-25.7) | 37.7 (32.3-43.1) | 18.9 (14.5-23.3) |
| **Total (n)** | Male | 1377 | 1739 | 2111 | 1148 | 771 | 422 | 1528 |
|  | Female | 1617 | 1273 | 3732 | 1050 | 650 | 318 | 2404 |
| **Total (%)** | Male | 15.1 | 19.1 | 23.2 | 12.6 | 8.5 | 4.6 | 16.8 |
|  | Female | 14.6 | 11.5 | 33.8 | 9.5 | 5.9 | 2.9 | 21.8 |
